# Hybrid MM/CG Webserver: Automatic Set Up of Molecular Mechanics/Coarse-Grained Simulations for Human G Protein-Coupled Receptor/Ligand Complexes

**DOI:** 10.3389/fmolb.2020.576689

**Published:** 2020-09-04

**Authors:** Jakob Schneider, Rui Ribeiro, Mercedes Alfonso-Prieto, Paolo Carloni, Alejandro Giorgetti

**Affiliations:** ^1^Computational Biomedicine, Institute for Advanced Simulation IAS-5/Institute for Neuroscience and Medicine INM-9, Forschungszentrum Jülich GmbH, Jülich, Germany; ^2^JARA-Institute: Molecular Neuroscience and Neuroimaging, Institute for Neuroscience and Medicine INM-11/JARA-BRAIN Institute JBI-2, Forschungszentrum Jülich GmbH, Jülich, Germany; ^3^Department of Physics, RWTH Aachen University, Aachen, Germany; ^4^Department of Biotechnology, University of Verona, Verona, Italy; ^5^Medical Faculty, Cécile and Oskar Vogt Institute for Brain Research, Heinrich Heine University Düsseldorf, Düsseldorf, Germany

**Keywords:** MM/CG, molecular mechanics, coarse-grained, hybrid methods, webserver, G protein-coupled receptor, ligand, molecular dynamics simulation

## Abstract

Hybrid Molecular Mechanics/Coarse-Grained (MM/CG) simulations help predict ligand poses in human G protein-coupled receptors (hGPCRs), the most important protein superfamily for pharmacological applications. This approach allows the description of the ligand, the binding cavity, and the surrounding water molecules at atomistic resolution, while coarse-graining the rest of the receptor. Here, we present the Hybrid MM/CG Webserver (mmcg.grs.kfa-juelich.de) that automatizes and speeds up the MM/CG simulation setup of hGPCR/ligand complexes. Initial structures for such complexes can be easily and efficiently generated with other webservers. The Hybrid MM/CG server also allows for equilibration of the systems, either fully automatically or interactively. The results are visualized online (using both interactive 3D visualizations and analysis plots), helping the user identify possible issues and modify the setup parameters accordingly. Furthermore, the prepared system can be downloaded and the simulation continued locally.

## Introduction

**FIGURE 1:**
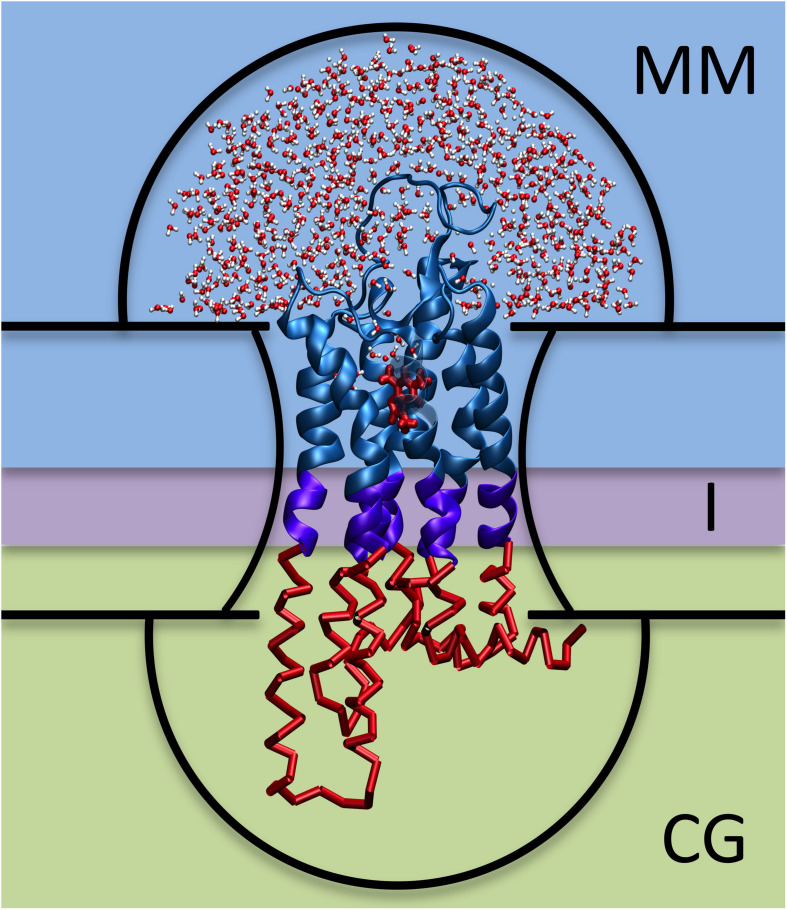
Hybrid MM/CG scheme. The three regions (MM, I, and CG) are framed with different background colors and the five potential walls (upper and lower hemispheres, upper and lower membrane planes, and membrane surface) are indicated with black lines.

Human G protein-coupled receptors (hGPCRs) are the largest drug target superfamily (Hauser et al., 2017). One third of FDA-approved drugs target ∼14% hGPCRs (Hauser et al., 2018) and this protein class has a further, untapped pharmacological potential. Unfortunately, rational drug design is hampered by the lack of experimental structures for more than 90% hGPCRs^1^ (Munk et al., 2019; Qu et al., 2020). Structural insights into ligand poses for these cases can be obtained by computational modeling. Reliable predictions can be made by docking approaches on homology models, based on templates sharing overall sequence identity >35–40% and/or high conservation of binding site residues (Beuming and Sherman, 2012; Kufareva et al., 2014). For lower resolution models, however, the uncertainty in the structure (and particularly in the orientation of side chains) decreases the accuracy of the docking predictions and, thus, follow-up molecular dynamics (MD) simulations are recommended (Kufareva et al., 2014; Cavasotto and Palomba, 2015; Esguerra et al., 2016; Heifetz et al., 2016; Fierro et al., 2017; Lupala et al., 2018; Rodríguez-Espigares et al., 2020). In an effort at addressing this issue, we have developed a Hybrid Molecular Mechanics/Coarse-Grained (MM/CG) simulation approach (Neri et al., 2005, 2008; Leguèbe et al., 2012; Marchiori et al., 2013; Sandal et al., 2015; Capaldi et al., 2018; Alfonso-Prieto et al., 2019; Fierro et al., 2019). The receptor/ligand interactions are described in atomistic detail, including explicit water molecules in the binding site (MM region), while the rest of the receptor is coarse-grained (CG region) (Schneider et al., 2018). The all-atom force fields used (Schneider et al., 2020) for the MM part of the protein and water are the Amber14SB (Maier et al., 2015) and TIP3P (Jorgensen et al., 1983), respectively, whereas the ligand can be described using either GAFF or GAFF2 (Wang et al., 2004; Case et al., 2020). The CG region is described by a Gō-like (Gō and Abe, 1981) potential. A region at the interface between the MM and CG parts couples the two levels of resolution (Figure 1). The membrane is described implicitly by introducing five potential walls (Leguèbe et al., 2012; Schneider et al., 2018). Two planar walls coincide with the height of the head groups of the membrane lipids, two hemispheric walls cap the extracellular and intracellular ends of the protein and prevent water evaporation, and the last wall follows the initial shape of the interface between protein and membrane, mimicking the effect of the lipid acyl tails (Figure 1). The scheme can be used also for GPCRs other than those from *Homo sapiens.* This approach turned out to be able to reproduce the ligand poses for four different hGPCRs (Schneider et al., 2020). These include the adenosine 2A receptor in complex with caffeine (i.e., the example case of the webserver), the human bitter receptor 16 in complex with phenyl-β-D-glucopyranoside, the β2-adrenergic receptor with adrenaline, and the dopamine D3 receptor with eticlopride. The structures of these hGPCR/ligand complexes were either experimentally determined, taken from all-atom MD trajectories or predicted based on templates with decreasing resolution, up to extremely low sequence identity (<15%) (Schneider et al., 2020). Retrospective validation against available X-ray structures and mutagenesis data confirmed that the MM/CG approach can predict correct ligand poses and identify experimentally determined binding residues^2^ (Schneider et al., 2020), regardless of the model resolution. In addition, the MM/CG simulations can provide insights into the flexibility of receptor–ligand interactions and hydration of the binding cavity, at a lower computational cost than all-atom molecular dynamics simulations.

The increasing number of applications and requests to use the MM/CG approach has prompted us to develop the Hybrid MM/CG webserver, a publicly accessible web interface aimed at preparing and running short MM/CG simulations of hGPCR/ligand complexes (available since December 2019). To the best of our knowledge, this is the only webserver dedicated to this task. It complements other excellent online resources (Table 1) aimed at preparing all-atom or coarse-grained molecular dynamics (MD) simulations of GPCRs and GPCR/ligand complexes (or in general membrane proteins).

**TABLE 1:** Available online resources for MD simulations of GPCRs.

Name	Functionality	System Setup				MD simulations
		Structural modeling	Membrane inclusion	Ligand inclusion	Force field used	
GPCR-ModSim (Esguerra et al., 2016)	Web-interactive system setup/simulation	✓^a^	✓	**∼**^b^	MM	✓ (AA, 5ns)
CHARMM-GUI (Jo et al., 2008; Lee et al., 2016)	Web-interactive system setup	**×**	✓	∼^c^	MM, CG	**×**
MERMAID (Damre et al., 2019)	Web-interactive system setup/simulation	**×**	✓	**×**	CG	✓ (CG, 100ns)
Hybrid MM/CG (This work)	Web-interactive system setup/simulation	∼^d^	✓ (implicit)	✓	MM/CG	✓ (MM/CG, 10ns)
PACKMOL-Memgen (Schott-Verdugo and Gohlke, 2019)	Local command line system setup	**×**	✓	∼^e^	MM	**×**
GPCRmd (Rodríguez-Espigares et al., 2020)	Database of precomputed MD simulations	**×**	✓	✓	MM	✓ (AA)^f^
MemProtMD (Newport et al., 2019)	Database of precomputed MD simulations	**×**	✓	**✓**	MM, CG	∼ (CG; AA)^g^

*AA, all-atom; CG, coarse-grained; MM, molecular mechanics. ^*a*^Homology modeling of GPCRs only; docking must be performed externally. ^*b*^Ligands should already be included in the OPLS-AA force field or parameterized externally. ^*c*^Although the Membrane Builder tool does not offer the option to include ligands, ligand parameterization can be accomplished using the Ligand Reader and Modeler tool also available in the CHARMM-GUI webserver. ^*d*^Homology modeling and ligand docking can be performed with other available webservers, including the linked GOMoDo webserver. ^*e*^Packmol-Memgen allows to keep ligands of interest but they have to be parameterized externally using other AmberTools. ^*f*^All set up and trajectory files can be downloaded. Simulations of the GPCRmd dataset typically include three replicas, 0.5 μs each, whereas simulations of the so-called “Individual contributions” section can vary in the number of replicas and simulation length. ^*g*^Trajectory files of the 1 μs CG assembly simulations cannot be downloaded. However, an equilibrated CG snapshot and the corresponding backmapped AA structure, together with the files needed to run subsequent CG or AA simulations, respectively, are available for download.*

The Hybrid MM/CG webserver requires only the coordinates of the receptor/ligand complex (as PDB file). These may come from experimental structures, simulation snapshots, or computational models generated with other webservers, such as the ones listed in the “Input” section. Furthermore, we have linked our GOMoDo webserver^3^ for modeling and docking of GPCRs to the Hybrid MM/CG webserver, so that the complexes generated with GOMoDo can be directly transferred. The user is first guided through a set of user-friendly forms for preparing the setup files. The procedure requires only a few minutes. Then, the user runs the initial MM/CG simulation steps (up to 10 ns) directly on the server. The resulting files can be downloaded and the MM/CG simulation can be continued using local resources.

## Materials and Methods

### Input

The input PDB file can be obtained from experimental databases, such as the Protein Data Bank (Berman et al., 2000; Rose et al., 2016), GPCRdb (Pándy-Szekeres et al., 2018) or GPCRmd (Rodríguez-Espigares et al., 2020), as well as computational services like GOMoDo (Sandal et al., 2013), GPCR-ModSim (Esguerra et al., 2016), GPCR-SSFE (Worth et al., 2017), GPCRM (Miszta et al., 2018), Galaxy7TM (Lee and Seok, 2016), GPCRautomodel (Launay et al., 2012), @TOME (Pons and Labesse, 2009) and others (reviewed in Busato and Giorgetti, 2016). The GOMoDo webserver (Sandal et al., 2013), which can be used for homology modeling of hGPCRs and subsequent docking of ligands, is linked directly with the Hybrid MM/CG webserver. Several structures of the same hGPCR/ligand complex obtained under different membrane compositions and/or in different activation states can be funneled into the webserver to indirectly (and very approximately) account for the influence of explicit lipids and of large conformational changes of the receptor, respectively.

### Setup Modes

The Hybrid MM/CG webserver offers two system setup modes:

#### Automatic Preparation

Here, the user just needs to upload a PDB file of the hGPCR in complex with its ligand or transfer it directly from the GOMoDo webserver^3^ (Sandal et al., 2013). All simulation parameters are set according to default values that are defined in the documentation^4^.

#### Interactive Preparation

Using the interactive preparation method, the same steps as in the automatic preparation are carried out to set up the system. The advantage is that several parameters, such as the position of the interface between MM and CG regions and the size of the hemispheric potential walls (see Figure 1), can be adjusted. Furthermore, intermediate results can be visualized, such as the input structure, aligned orientation in membrane, solvation (water drop), level of coarse-graining, and position of the wall potentials. More details about the individual preparation steps and parameters are explained in the Documentation section of the webserver^4^.

### Workflow

**FIGURE 2:**
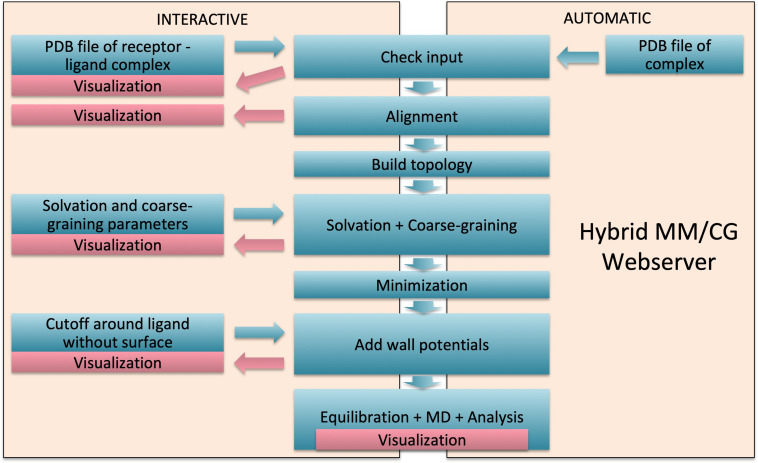
Webserver workflow. Interactive mode with manual intervention is presented on the left and fast automatic mode on the right.

**FIGURE 3:**
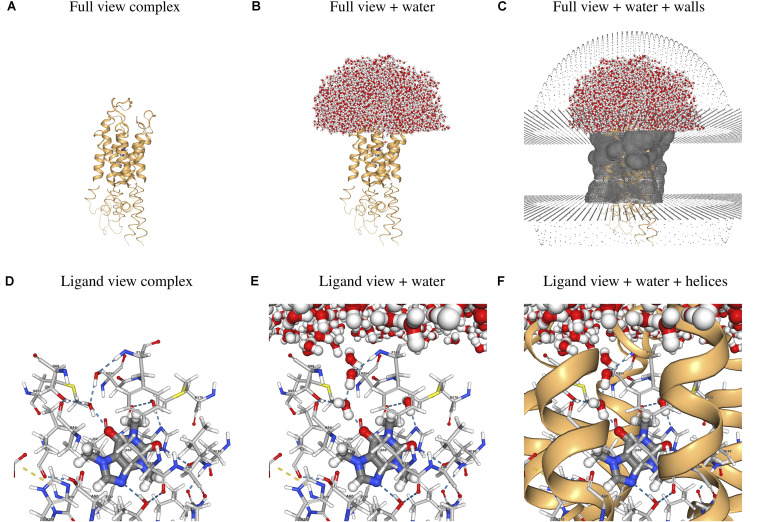
Sample 3D visualizations created by the webserver using as example the hA_2A_R/CFF complex. **(A–C)** Full view of the hGPCR/ligand complex and **(D–F)** close-up view of the ligand and its interactions with protein and water molecules. Images were generated with the screenshot tool in the NGL viewer (Jakalian et al., 2004) used in the “Results” section of the webserver. Atoms are colored using the default NGL color code and ligand interactions are determined using the default NGL definition.

The steps carried out by the webserver for MM/CG system preparation and short simulation (Figure 2) are the following:

• *File upload*. The preparation starts with a PDB file of the hGPCR/ligand complex. This file can be obtained from one of the databases and webservers mentioned in the “Input” section and uploaded by the user into the Hybrid MM/CG webserver. Alternatively, it can be transferred directly from GOMoDo (Sandal et al., 2013).
• *Check Input*. The input PDB file is checked for missing residues and the numbering is corrected if possible (i.e., no residues are missing). The ligand name is determined by comparing the list of residue names in the input PDB file and in the Amber14SB force field. Known amino acids and capping groups are discarded and the remaining residue name is considered to be the ligand.
• *Alignment*. The orientation and position of the receptor with respect to the hydrophobic core of the lipid bilayer are determined using the PPM tool (Lomize et al., 2012). The initial PDB file of the hGPCR/ligand complex is then superimposed to the PPM-aligned structure of the receptor using lovoalign (Martínez et al., 2007).
• *Build topology: Protein*. The program pdb2gmx (Berendsen et al., 1995; Hess et al., 2008) is used to build the receptor topology using the Amber14SB force field (Maier et al., 2015). Protonation states of receptor titratable residues are determined automatically by pdb2gmx. In case the user wants to bypass the automatic assignment, different protonation states can be enforced with minimal manual editing of the input PDB file by changing the corresponding residue name (e.g., from “HIS” to “HIP” for a doubly protonated histidine).
• *Build topology: Ligand.* Protonation of the ligand at pH = 7 is determined using OpenBabel (O’Boyle et al., 2011). The ligand topology is built with ACPYPE (Sousa da Silva and Vranken, 2012) and Antechamber (Wang et al., 2001), using AM1-BCC charges (Jakalian et al., 2004) and the general Amber force field. GAFF (Wang et al., 2004) is used in the automatic preparation, but the user can choose between GAFF and GAFF2 (Case et al., 2020) in the interactive preparation mode.
• *Solvation*. The simulation box is created and the system is solvated using the TIP3P (Jorgensen et al., 1983) force field. Water molecules below the upper membrane plane are deleted. Hemisphere sizes are defined (default radii: 50 Å) and water outside the upper hemisphere is deleted, so that only a water “drop” solvating the extracellular part of the receptor is kept (see Figure 1).
• *Coarse-graining.* The regions of different resolutions are defined by two cutoff values, one defining the boundary between the MM and interface regions (default is centered between the two membrane planes) and the other between the interface and CG regions (6 Å below the first cutoff). Coarse-graining is performed on residues below the second cutoff level using a Gō-like (Gō and Abe, 1981) model.
• *Minimization*. A simple minimization of the system is done using the steepest descent method until a maximum force of 1,000 kJ mol^–1^ nm^–1^ is reached.
• *Add wall potentials*. Wall potentials are added to the system according to the aforementioned heights and radii. Wall grid points that are too close to the ligand are deleted according to the chosen cutoff (default 7 Å).
• *Visualization.* The user is redirected to the “Results” section where preparation results can be inspected using interactive 3D visualizations and graphs.
• *Equilibration*. An optional short equilibration (2 ns) and short MD simulation (2 ns) can be started from the “Results” section as well.
• *MD*. The simulation can be extended up to 10 ns, in 2 ns increments, in order to offer the possibility to check the intermediate results.
• *Visualization.* The minimization, equilibration, and MD are analyzed automatically in terms of potential energy and temperature after completion of the individual step (see Supporting Information). Interactive 3D visualizations are available for the results of the preparation, equilibration, and MD (see Figure 3).

### Output

Results can be viewed and downloaded for 2 weeks (renewal possible) by bookmarking the link or alternatively by using the corresponding ID^5^. The full output of the preparation can be downloaded as a compressed archive file including the input, output, and log files of all preparation and simulation steps. The downloaded files can be used to continue the MM/CG simulations locally. Experienced users have the possibility to download the prepared system and tune the partial charges, as well as other parameters, before running the simulation on their local computer. The Download Code section of the server provides an installation script, which contains the links to the source code for both GROMACS 4.5.1 (from the GROMACS website) and the Hybrid MM/CG patch (from our server website).

### Server Architecture

#### Front-End

The web interface was developed with the DJANGO 3.0 Web framework^6^ and designed with the Bootstrap 4 front-end open-source toolkit^7^. Interactive 3D visualizations were implemented with the NGL library for molecular visualization (Rose and Hildebrand, 2015) and the interactive graphs were created using the Highcharts SVG-based JavaScript charting library^8^.

#### Back-End

For historical reasons, different programming and scripting languages are used for the workflow such as Bash, Python, Perl, Tcl, AWK, and JavaScript. Furthermore, different software packages are used in the preparation: a patched GROMACS 4.5.1 version (Berendsen et al., 1995; Hess et al., 2008), VMD 1.9.3 (Humphrey et al., 1996), PPM (Lomize et al., 2012), lovoalign (Martínez et al., 2007), ACPYPE Rev: 10101 (Sousa da Silva and Vranken, 2012), Antechamber (Wang et al., 2001) from AmberTools16 and OpenBabel 2.3.2 (O’Boyle et al., 2011). All parts were combined using the DJANGO 3.0 Web framework^6^.

## Results

As an application case, we considered the human adenosine 2A receptor (hA_2__A_R) in complex with its antagonist caffeine (CFF). CFF was proposed to have a protective and therapeutic effect against Parkinson’s disease (Chen et al., 2001; Sonsalla et al., 2012; Petzer and Petzer, 2015; Nazario et al., 2017). The input PDB file of the hA_2__A_R/CFF complex used for the Example can be downloaded from the same page. The demonstration can be run by simply starting the preparation workflow^9^ without uploading any PDB file. Precomputed results for this example case can be found on the server^10^ and selected parts of these results can be seen in Figure 3. Figures 3A–C show the whole hA_2__A_ receptor in complex with CFF, with and without water solvating the binding site and the extracellular loops, as well as the walls mimicking the membrane and preventing water evaporation. Figures 3D–F show the ligand and its direct and water-mediated interactions with residues in the binding cavity, as determined by the NGL viewer (Jakalian et al., 2004). The system can be inspected in 3D after the preparation, short equilibration, and production runs. In addition, plots showing the time evolution of the potential energy and temperature during the different simulation steps are generated (see Supplementary Figures 1–3).

## Conclusion

The hybrid MM/CG protocol has been successfully used to predict ligand poses in a variety of hGPCRs (Leguèbe et al., 2012; Marchiori et al., 2013; Sandal et al., 2015; Fierro et al., 2017; Capaldi et al., 2018; Fierro et al., 2019). Moreover, the Amber-based MM/CG poses improved significantly relative to those obtained by simple docking, especially for low resolution starting models (Schneider et al., 2020). However, the setup of such simulations has been time-consuming and system-dependent. The webserver presented here automatically prepares all files needed to start the equilibration, in a short time: the hA_2__A_R/CFF example requires only 6 min. An additional advantage of the Hybrid MM/CG webserver over most of the online services for simulation of GPCRs (Table 1) is the automatic ligand parameterization. Although other webservers can also include ligands (see Table 1), their parameterization usually has to be done externally. The results of the preparation steps can be checked on-the-fly (e.g., ligand structure and full solvation of extracellular loops). No additional software (except the patch for the GROMACS code, which is available for download^11^) is needed to extend the simulations locally. This makes the method useful for people acquainted with MD simulations and not necessarily familiar with every single MM/CG preparation step.

The server could prospectively be used for setting up MM/CG simulations of different ligands in complex with the same hGPCR. Capitalizing on the limited computational cost of the MM/CG approach, this could pave the way for low-throughput virtual screening efforts. Furthermore, comparative studies of hGPCRs in multiple states, e.g., active/inactive, can be accomplished by setting up several simulations starting from different initial structures. Modeling of allosteric effects is currently not possible, because of the use of the Gō-like model for the part of the receptor pointing toward the intracellular side. We also plan to integrate the Amber-based MM/CG approach used by the webserver with the recently developed open boundary MM/CG for grand canonical simulations (Tarenzi et al., 2017, 2019), aiming at calculating ligand binding free energies. Further planned developments include the extension to other MD codes, the implementation of the CHARMM36m force field (Huang et al., 2017) for the MM/I regions, as well as the adaptation of the scheme to other membrane protein families, such as ion channels.

## Data Availability Statement

The Hybrid MM/CG Webserver, available at https://mmcg.grs.kfa-juelich.de/, is free and open to all. There is no login requirement.

## Author Contributions

JS, MA-P, PC, and AG contributed to workflow design and documentation. JS developed the server. RR designed the interface on the GOMoDo webserver. JS, RR, MA-P, and AG tested the webserver. All authors participated in writing the article.

## Conflict of Interest

The authors declare that the research was conducted in the absence of any commercial or financial relationships that could be construed as a potential conflict of interest.

## Footnotes

1 https://gpcrdb.org/structure/statistics as of June 2020.

2 the residues whose mutation is known experimentally to affect binding

3 gomodo.grs.kfa-juelich.de

4 mmcg.grs.kfa-juelich.de/documentation

5 mmcg.grs.kfa-juelich.de/results/+ID

6 djangoproject.com

7 getbootstrap.com

8 highcharts.com

9 https://mmcg.grs.kfa-juelich.de/

10 mmcg.grs.kfa-juelich.de/example_results/

11 mmcg.grs.kfa-juelich.de/download/install

## Abbreviations

AA: all-atom
CFF: caffeine
CG: coarse-grained
FDA: United States Food and Drug Administration
hA_2A_R: human adenosine 2A receptor
hGPCRs: human G protein-coupled receptors
MD: molecular dynamics
MM: molecular mechanics
MM/CG: molecular mechanics/coarse-grained.
