# Supplementary material for: Hybrid MM/CG Webserver: Automatic Set Up of Molecular Mechanics/Coarse-Grained Simulations for Human G Protein-Coupled Receptor/Ligand Complexes
Source: Front Mol Biosci. 2020 Sep 4;7:576689. doi: 10.3389/fmolb.2020.576689 (PMC7500467; doi:10.3389/fmolb.2020.576689)
Supplement: Supplementary file 1 [file Data_Sheet_1.PDF]

Supplementary Material

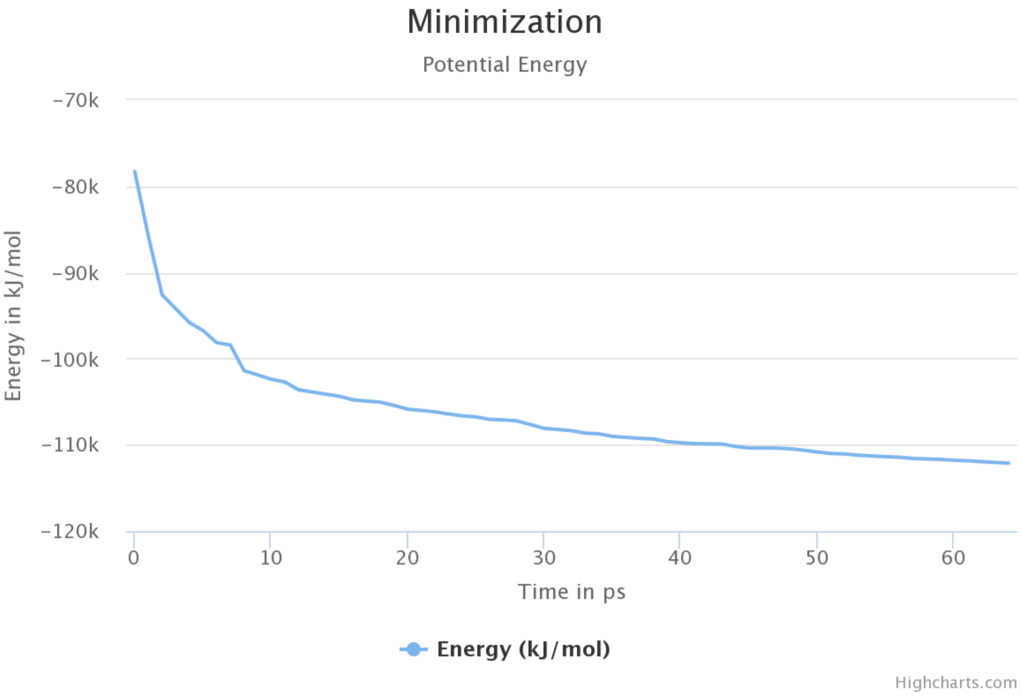

Supplementary Figure 1. Potential energy plot of the minimization created by the Hybrid MM/CG webserver using the $hA_{2A}R$/CFF complex. The image was generated with the interactive Highcharts[1] charting library used in the Results section of the webserver.

[1] highcharts.com

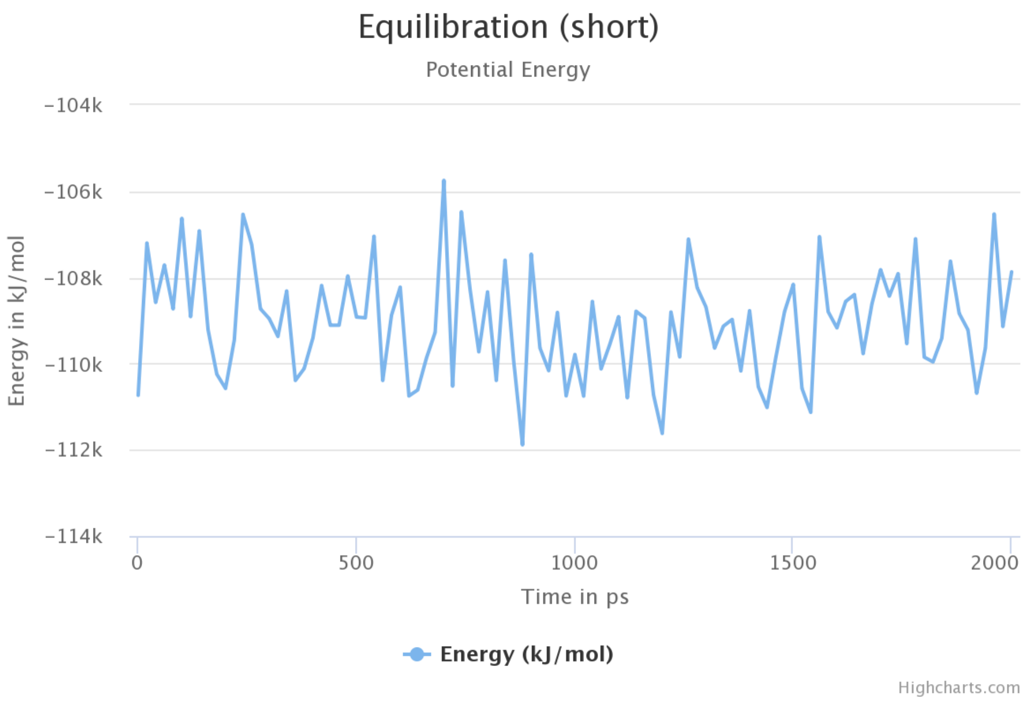

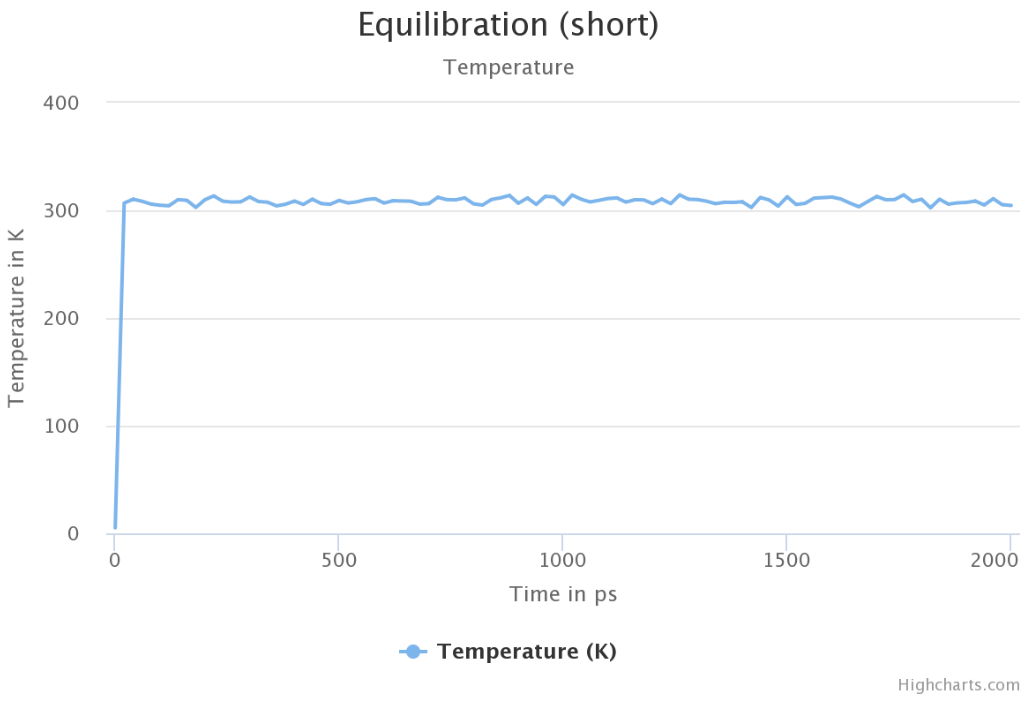

Supplementary Figure 2. Potential energy (above) and temperature (below) plots as a function of simulated time of the $hA_{2A}R$/CFF complex (equilibration phase). The plots have been created by the Hybrid MM/CG webserver.

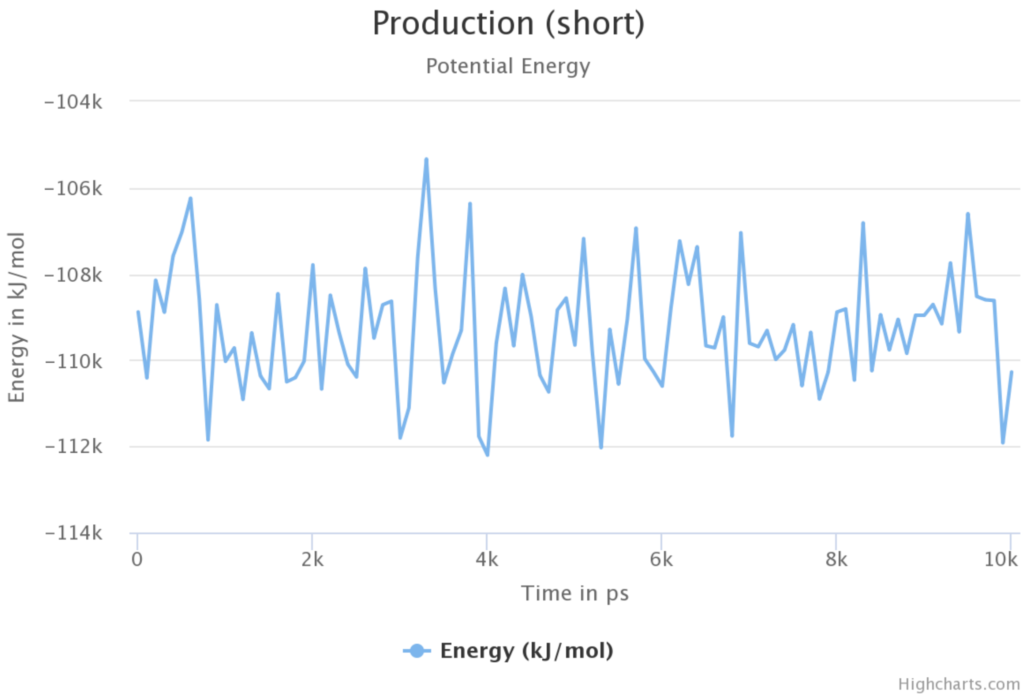

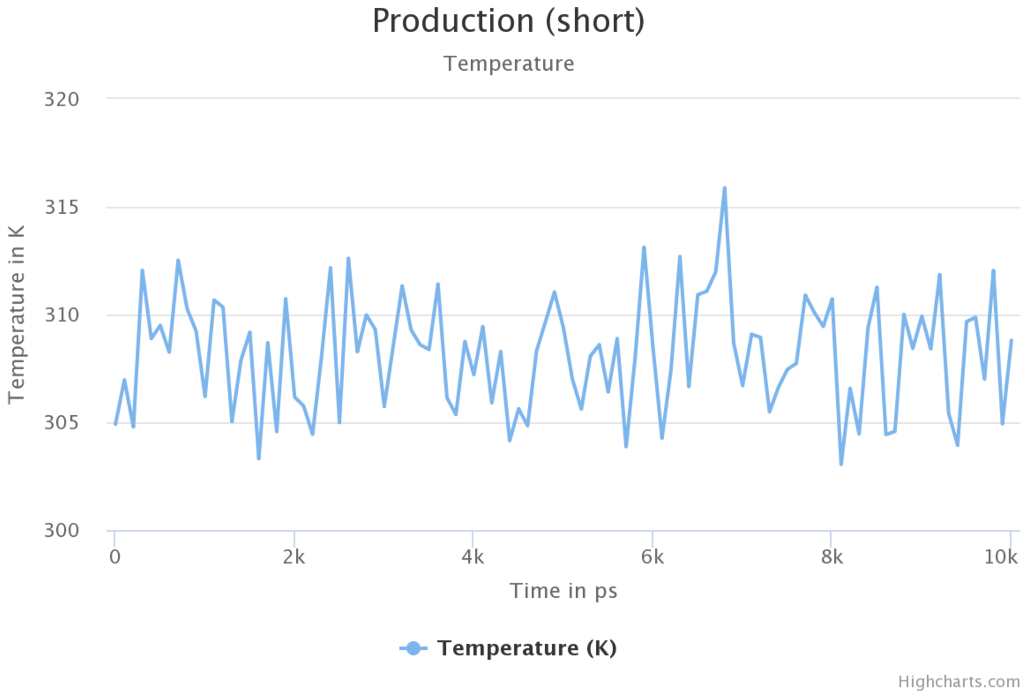

Supplementary Figure 3. Potential energy (above) and temperature (below) plots as a function of simulated time of the $hA_{2A}R$/CFF complex (production run). The plots have been created by the Hybrid MM/CG webserver.
